# Role of PGE_2_ in colonic motility: PGE_2_ attenuates spontaneous contractions of circular smooth muscle via EP_4_ receptors in the rat colon

**DOI:** 10.1186/s12576-021-00791-4

**Published:** 2021-02-23

**Authors:** Shin-Ichiro Karaki, Ryo Tanaka

**Affiliations:** 1 Laboratory of Physiology, Department of Environmental Life Sciences, University of Shizuoka, 52-1 Yada, Suruga-ku, Shizuoka, 422-8526 Japan; 2 Testing and Research Laboratories, HAMRI Co., Ltd., 2654-3 Osaki, Koga, Ibaraki 306-0101 Japan

**Keywords:** Colonic motility, Giant migrating contraction, Giant contraction, Prostaglandin, EP receptor

## Abstract

Colonic motor activity is important for the formation and propulsion of feces. The production of prostaglandins (PGs) in colonic tissue is considered to play a critical role in the generation and regulation of colonic motility. In this study, we investigated the inhibitory effects of PGE_2_ and selective agonists of four EP receptors on the spontaneous phasic contractions, called ‘giant contractions’ (GCs), of mucosa-free circular smooth muscle strips from the rat middle colon. Neural blockade with tetrodotoxin (TTX) increased the frequency and amplitude of the GCs by about twofold. However, inhibiting PG production with piroxicam reduced the GC frequency in the presence of TTX, but did not affect the GC amplitude. In the presence of both TTX and piroxicam, exogenous PGE_2_ and each EP receptor agonist were cumulatively added to the tissue bath. In this setting, PGE_2_, the EP_2_ agonist ONO-AE1-259, and the EP_4_ agonist ONO-AE1-329, but not the EP_1_ agonist ONO-AE-DI-004 or the EP_3_ agonist ONO-AE-248, concentration-dependently reduced the GC frequency and amplitude. The PGE_2_-induced inhibition of GC frequency and amplitude was inhibited by the EP_4_ antagonist ONO-AE3-208, but not by the EP_1/2_ antagonist AH6809. Immunohistochemistry revealed the EP_2_ and EP_4_ receptors were localized in perinuclear sites in circular smooth muscle cells. EP_2_ immunoreactivity was also located in GFAP-immunoreactive enteroglia, whereas EP_4_ immunoreactivity was also located in HU (embryonic lethal, abnormal vision [ELAV] protein; a marker of all myenteric neurons)-immunoreactive myenteric nerve cell bodies. These results suggest that the PGs produced in the colonic tissue inhibit the GC frequency and amplitude of circular muscle in the rat middle colon, and is mediated by EP_4_ receptors expressed in the smooth muscle cells.

## Introduction

Colonic motor activity must be precisely regulated to form and propel feces adequately. To perform these functions, the colonic wall displays complex and varied spatiotemporal characteristics of motility. In a variety of species, including dogs, humans, and rats, three distinct types of contractions have been characterized by in vivo recordings: rhythmic phasic contractions (RPCs), giant migrating contractions (GMCs), and tonic contractions (TCs) [1].

The GMCs, also called ‘mass movements’ or ‘mass peristalsis’, occur once or twice a day in the human and dog colon, but occur very frequently in the rat colon, with a reported frequency of 44.1 ± 6.0 (proximal), 34.5 ± 6.1 (middle), and 16.9 ± 3.0 (distal colon) contractions per hour (mean ± standard error of the mean [SEM], *n* = 6) [2]. Although isolated circular muscle (CM) strips from the human and dog colon only generate RPCs and TCs in vitro, giant concentrations (GCs) similar to GMCs have been recorded in isolated rat CM strips from the middle colon [3]. Therefore, smooth muscle strips from the rat colon are considered a useful material to investigate the mechanisms underlying the generation and regulation of GMCs.

The motility status of the colon is thought to be induced and regulated by myogenic and neurogenic mechanisms involving the network of the interstitial cells of Cajal (ICC), the nervous system, the endocrine system, and the immune system [4, 5]. Prostaglandins (PGs) are one of the most important chemical mediators of motility, and are ubiquitously produced in almost all tissues of the body and affect a variety of physiological functions, including gastrointestinal (GI) motility. In the 1960s, before the PG receptors were identified, it was reported that PGE_2_ enhances the motility of longitudinal muscle (LM) [6–8] but reduces the motility of CM [7, 8]. In the 1990s, PGE_2_ receptors were identified as G-protein-coupled receptors and subdivided into four subtypes, EP_1_, EP_2_, EP_3_, and EP_4_, in humans [9–11], mice [9, 12–15], and rats [16]. The EP_1_ receptor is coupled to the G_q_ protein and increases intercellular Ca^2+^ concentrations. The EP_2_ and EP_4_ receptors are coupled to G_s_ and activate adenylyl cyclase to produce cyclic adenosine monophosphate (cAMP). The EP_3_ receptor is coupled to G_i_ and reduces intracellular cAMP levels [17].

In our previous study, we reported that in rat middle colonic LM strips, PG production is required for the generation of spontaneous GCs because the cyclooxygenase (COX) inhibitor, piroxicam, completely inhibits GCs [18]. Moreover, GC generation is mediated by the EP_1_ and EP_3_ receptors expressed in LM cells [18]. In contrast, Martines-Cutillas et al*.* [19] reported that the PGE_2_-induced reduction of CM motility (area under the curve [AUC] of contractions) in the murine colon is mediated by postjunctional EP_2_ and EP_4_ receptors. In the present study, we investigated the involvement of neural activity and PG production in the GCs of rat middle colonic CM strips, examining the frequency and amplitude of the GCs separately. The aim of this study was to determine the role of PGs in colonic motility and to identify the EP receptor subtype(s) involved in the frequency and amplitude of GCs.

## Materials and methods

### Animal and tissue preparation

Male Wistar rats (288.7 ± 6.4 g, *n* = 32; Japan SLC, Hamamatsu, Japan) were anesthetized by inhalation of isoflurane anesthetic, and were decapitated with a guillotine. Animal handling and euthanization were performed in accordance with the *Guidelines for the Care and Use of Laboratory Animals of the University of Shizuoka*, and the study was approved by the University of Shizuoka Animal Use Ethics Committee (Shizuoka, Japan). Segments of the middle colon were removed and immediately immersed in ice-cold Krebs–Ringer solution (in mmol L^−1^: 117 NaCl, 4.7 KCl, 1.2 MgCl_2_, 1.2 NaH_2_PO_4_, 25 NaHCO_3_, 2.5 CaCl_2_, and 11 glucose) saturated with 95% O_2_ and 5% CO_2_. The segments were then cut open along the mesenteric border and pinned mucosal-side-up on a silicone rubber-coated Petri dish filled with cold Krebs–Ringer solution. The mucosa and submucosa were removed from the tissues with sharp forceps under a stereomicroscope, and the tissues were cut parallel to the circular axis of CM to produce the CM strip preparations (approximately 2 mm wide and 8–10 cm long). One end of each preparation was connected to an isometric force transducer (type 45196A; NEC San-ei Instruments, Ltd, Tokyo, Japan) with a surgical thread and the other end was fixed to a supporting rod with a thread. The preparations were suspended in tissue baths (Radnoti, Monrovia, CA, USA) containing 15 mL of Krebs–Ringer solution at 37 °C, which was continuously bubbled with 95% O_2_ and 5% CO_2_ during the experiments. After the tissue preparations were set up, they were equilibrated for about 1–3 h until spontaneous GCs occurred stably for 20 min. In all experiments, carbachol (CCh; 10^−5^ M) was added in the final stage to normalize the amplitude of the GCs.

### Experimental protocols and data analysis

*Mean frequency and amplitude of GCs* The mean frequency and amplitude of the GCs were determined as follows. All amplitudes (mN) and the times of the peaks of GCs were recorded over a period of 20 min. The average amplitude was calculated, and the % amplitude of a CCh (10^−5^ M)-evoked contraction in the same tissue was determined as the mean amplitude (%CCh). The average period (min) was calculated, and 1/(average period) was taken as the mean frequency (GCs min^−1^).

**Fig. 1:**
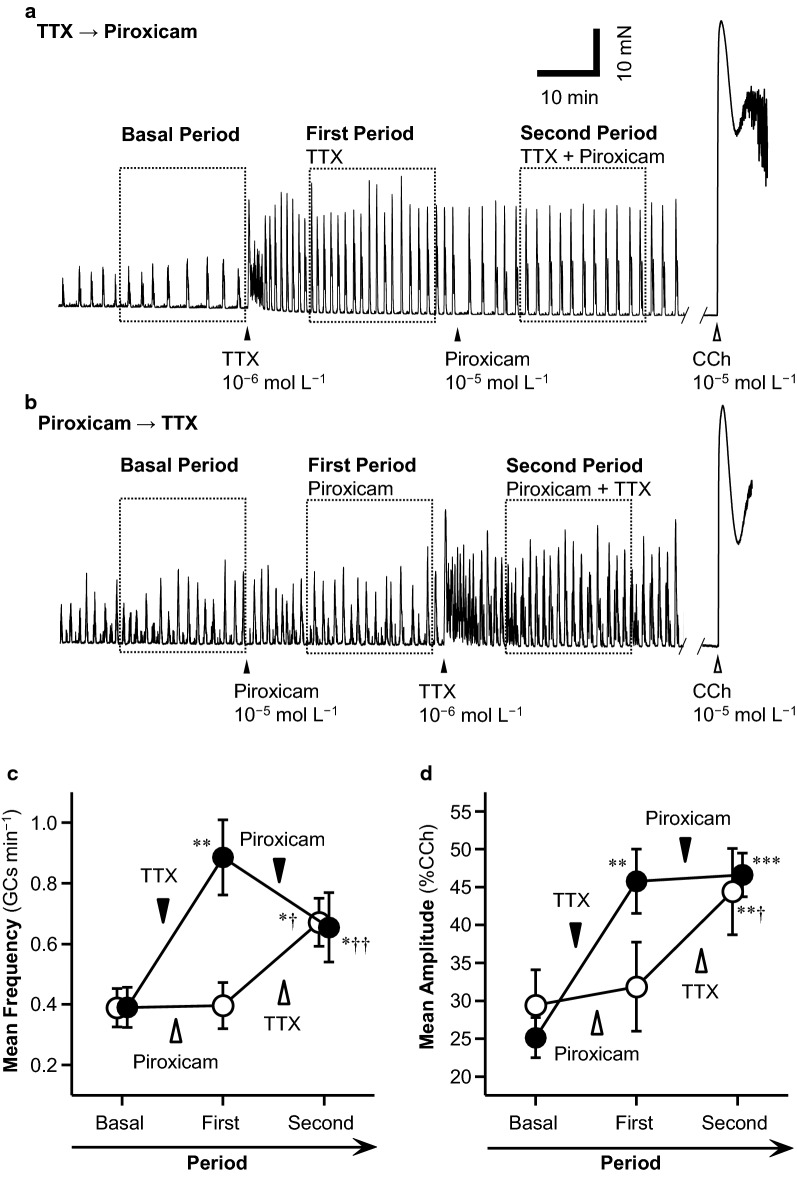
Effects of neural activity and prostaglandin (PG) production on the giant contractions (GCs) of circular muscle (CM) in the rat middle colon. After equilibration, spontaneous GCs were observed and basal GCs recorded (**a** and **b**, basal period). The neural blocker TTX (10^−6^ mol L^−1^;  **a**) or the COX inhibitor piroxicam (10^−6^ mol L^−1^; **b**) was then added to the organ bath, and from 10 min after its addition, the GCs in the presence of TTX (**a**) or piroxicam (**b**) were recorded for 20 min as the first period. Piroxicam (**a**) or TTX (**b**) was then added, and from 10 min after its addition, GCs were recorded for 20 min as the second period. Finally, CCh was added to the organ bath to normalize the GC amplitude. Representative traces of GCs during TTX → piroxicam treatment (**a**) and piroxicam → TTX treatment (**b**), and changes in the mean frequencies (**c**) and amplitudes (**d**) are expressed as means ± SE (TTX → piroxicam, *n* = 8; piroxicam → TTX, *n* = 6). *P < 0.05, **P < 0.01, and ***P < 0.001 indicate significant differences from the basal values, and †*P* < 0.05 and ††*P* < 0.01 indicate significant differences from the first period values, by paired *t* test with Holm’s correction

*Involvement of neural activity and prostaglandin production in spontaneous GCs* To investigate the contributions of neural activity and endogenous PG production to spontaneous GCs, a neural blocker, tetrodotoxin (TTX; 10^−6^ M), was first added, and the mean frequency and amplitude of the GCs were determined in two periods: for 20 min immediately before the addition of TTX (basal period), and for 20 min extending from 10 to 30 min after the addition of TTX (first period). At 30 min after the addition of TTX, the COX inhibitor piroxicam (10^−5^ M) was added, and the mean frequency and amplitude of the GCs were determined for 20 min from 10 to 30 min after the addition of piroxicam (second period) (Fig. 1a). An experiment in which the compounds were added in a different order was also performed: piroxicam was added first, followed 30 min later by TTX (Fig. 1b).

*Concentration–response analysis of PGE*_*2*_* and each EP receptor agonist* After the experiments described above and in the presence of TTX (10^−6^ M) and piroxicam (10^−5^ M), PGE_2_, ONO-DI-004 (EP_1_ agonist), ONO-AE1-259 (EP_2_ agonist), ONO-AE-248 (EP_3_ agonist), or ONO-AE1-329 (EP_4_ agonist) was added at 30-min intervals to final concentrations ranging from 10^−11^ to 10^−6^ mol L^−1^ or until the GCs disappeared. The mean frequencies and amplitudes of the GCs were determined for 20 min immediately before the first addition of each agonist (basal), and for 20 min from 10 to 30 min after the addition of each concentration of each agonist. The effects of each concentration of each agonist were calculated as the % mean frequency and amplitude relative to the basal mean frequency and amplitude, respectively.

**Fig. 2:**
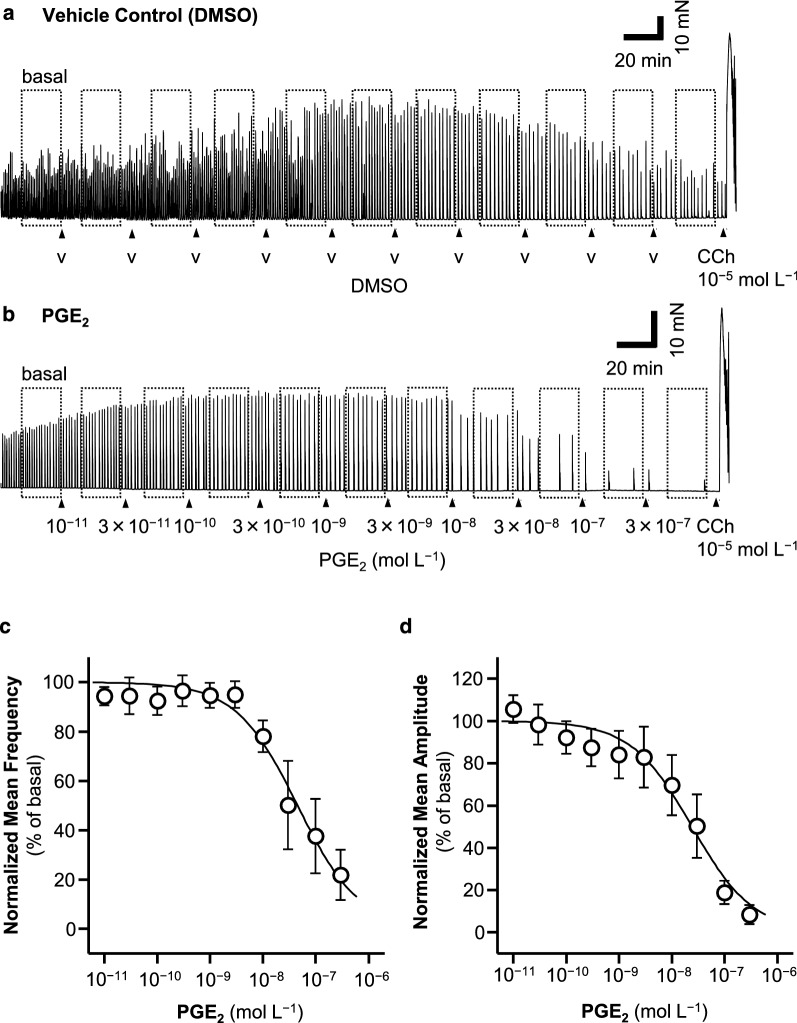
Concentration-dependent inhibitory effects of PGE_2_ on GC frequency and amplitude. In the presence of TTX and piroxicam, vehicle control (DMSO) or PGE_2_ was added cumulatively to the bathing solution (10^−11^ to 3 × 10^−6^ mol L^−1^) in  ~ 30-min intervals. Representative traces after the addition of DMSO (**a**) or PGE_2_ (**b**) are shown. Concentration–response curves of the normalized mean frequency and amplitude (see Methods and Results) are shown in **c** and **d**. Data are expressed as means ± SE (*n* = 4)

Even when the tissues were treated only with vehicle solution (dimethyl sulfoxide [DMSO], 15 µL) at 30-min intervals, the frequency and amplitude of the GCs during the experiment changed with time, as shown in Fig. 2a and Additional file 1: Fig. S1. The % mean frequency decreased gradually for several hours (Fig. 2a and Additional file 1: Fig. S1A), and the % mean amplitude increased gradually for approximately the first 2 h, then gradually decreased (Fig. 2a and Additional file 1: Fig. S1B). Therefore, vehicle control experiments using tissue isolated from the same animal were included in each experiment to normalize the experimental data. The values were normalized to the % mean frequency and amplitude of the vehicle control values for the same period: (experimental value/vehicle control value) × 100.

Concentration–response curves for PGE_2_, ONO-AE1-259, and ONO-AE1-329 were fitted to the modified Hill equation with a nonlinear square procedure using Marquardt’s method, as follows:

$Normalized \% mean value =100\mathrm{\% }\times \left\{1-\frac{1}{1+{\left(\frac{{IC}_{50}}{[\mathrm{Agonist}]}\right)}^{{n}_{\mathrm{H}}}}\right\},$where IC_50_ is the 50% inhibitory concentration, [Agonist] is the concentration of the agonist, and *n*_H_ is the Hill coefficient.

*Analysis of the effects of EP receptor antagonists on the PGE*_*2*_*-evoked response* In the presence of TTX (10^−6^ mol L^−1^) and piroxicam (10^−5^ mol L^−1^), the mean frequencies and amplitudes of the GCs before and after the addition of AH6809 (EP_1_ and EP_2_ antagonist, 10^−5^ mol L^−1^), ONO-AE3-208 (EP_4_ antagonist, 10^−7^ mol L^−1^), or DMSO (vehicle control) were determined. PGE_2_ was then cumulatively added at 10^−8^, 3 × 10^−8^, 10^−7^, and 3 × 10^−7^ mol L^−1^ at 30-min intervals. The concentration–response curves for PGE_2_ were fitted with nonlinear least squares regression using KyPlot software (KyensLab Inc., Tokyo, Japan).

### Statistical analysis

All data are expressed as means ± SE. Statistical differences in the time course of changes before and after each treatment were tested with paired Student’s *t* test with Holm’s correction to obtain *P* values [20]. Statistical differences among groups in the mean frequencies and amplitudes of GCs at each concentration of PGE_2_ were tested with an unpaired *t* test and Holm’s correction to obtain *P* values.

### Chemicals

Carbachol and piroxicam were purchased from Sigma-Aldrich (St. Louis, MO, USA). TTX was from Tocris Bioscience (Ellisville, MO, USA). PGE_2_ was from Cayman Chemical (Ann Arbor, MI, USA). The EP agonists and antagonists were a kind gift from Ono Pharmaceutical (Osaka, Japan). The PGE_2_, piroxicam, EP agonists, and EP antagonists were dissolved in DMSO, and all other chemicals were dissolved in distilled water. The *K*i values of the EP agonists and antagonists for the EP receptor subtypes are shown in Table 1.

**Table 1:** *K*i values for EP receptor subtype-specific agonists and antagonists used in the present study

	*K*i [mol L^−1^]			
	EP_1_	EP_2_	EP_3_	EP_4_
Agonist				
PGE_2_ (endogenous)	2.2 × 10^−8^	6.8 × 10^−9^	9 × 10^−10^	1.1 × 10^−9^
ONO-DI-004 (EP_1_)	1.5 × 10^−7^	> 10^−5^	> 10^−5^	> 10^−5^
ONO-AE1-259 (EP_2_)	> 10^−5^	3 × 10^−9^	> 10^−5^	6.0 × 10^−6^
ONO-AE-248 (EP_3_)	> 10^−5^	3.7 × 10^−6^	7.5 × 10^−9^	4.2 × 10^−6^
ONO-AE1-329 (EP_4_)	> 10^−5^	2.1 × 10^−6^	1.2 × 10^−6^	9.7 × 10^−9^
Antagonist				
AH6809 (EP_1_/EP_2_)	1.3 × 10^−6^	5.22 × 10^−7^	> 10^−5^	> 10^−5^
ONO-AE3-208 (EP_4_)	> 10^−5^	> 10^−5^	3 × 10^−8^	1.3 × 10^−9^

PGE_2_ and AH6809 (rat) [16]

ONO-DI-004, ONO-AE1-259, ONO-AE-248, and ONO-AE1-329 (mouse) [31]

ONO-AE3-208 (mouse) [32]

### Immunohistochemistry

Tissues from the middle colons isolated from rats were immersed in phosphate-buffered saline (PBS) containing nicardipine (10^−5^ mol L^−1^) for several minutes to relax the smooth muscle, and the luminal contents were gently removed. The tissues were then rapidly frozen with optimal cutting temperature (OCT) compound (Sakura Finetek Japan, Tokyo, Japan) in liquid nitrogen, and stored at − 80 °C until use. The frozen tissues were cut into sections (10 µm thick) with a cryostat (CM1100, Leica Microsystems GmbH, Wetzlar, Germany), and immediately immersed in cold (− 20 °C) methanol for 10 min or Zamboni fixative (4 °C) for 1 h to fix the tissues. The fixed tissues were washed three times with PBS for 10 min each, and immersed in blocking solution (10% normal donkey serum, 1% Triton X-100, 0.5% bovine serum albumin, and 0.1% sodium azide in PBS) at room temperature for 30 min. After blocking, the tissues were incubated at 4 °C with the diluted primary antibodies shown in Table 2. After immunoreaction with the primary antibodies overnight, the tissues were washed three times with PBS for 10 min each and incubated at room temperature with the diluted secondary antibodies shown in Table 2, together with 4′,6-diamidino-2-phenylindole (DAPI; 5 µg mL^−1^). After immunoreaction for 1 h, the tissues were washed three times with PBS for 10 min each, and cover-slipped with Dako Fluorescence Mounting Medium (DakoCytomation, Glostrup, Denmark). The immunoreactivity and DAPI fluorescence in the tissues were visualized with a fluorescence microscope (IX70, Olympus, Tokyo, Japan), and the images were captured with a cooled charge-coupled device digital camera system (AxioVision 135, Zeiss, Munich- Hallbergmoos, Germany).

**Table 2:** Antibodies

Antigen	Host	Label	Dilution	Source
Primary antibody				
EP_2_ receptor	Rabbit		1:100	Acris Antibodies
EP_4_ receptor	Rabbit		1:100	Cayman
α-smooth muscle actin	Mouse		1:500	Sigma-Aldrich
HU				
GFAP	Mouse		1:2000	Chemicon
Secondary antibody				
Rabbit IgG	Donkey	Alexa Fluor 594	1:200	Molecular Probes
Mouse IgG	Goat	Alexa Fluor 488	1:200	Molecular Probes
Goat IgG	Donkey	Alexa Fluor 488	1:200	Molecular Probes

## Results

### Effects of TTX and piroxicam on basal frequencies and amplitudes of spontaneous GCs

After equilibration for about 1 h after the tissue was mounted to the force transducer, the spontaneous GCs were observed and recorded as the basal GCs (Fig. 1a and b, basal period). The addition of the neural blocker TTX (10^−6^ mol L^−1^) to the bathing solution evoked a transient burst increase in the frequency of GCs for 5–10 min (Fig. 1a), and the mean frequency then increased continuously from 0.39 ± 0.07 GCs min^−1^ (basal) to 0.89 ± 0.12 GCs min^−1^ (**P < 0.01; Fig. 1a and c) and the mean amplitude of the GCs increased from 25.1 ± 2.7% CCh (basal) to 45.8 ± 4.3% CCh (**P < 0.01 by paired *t* test with Holm’s correction, *n* = 8; Fig. 1a and d). The subsequent addition of the COX inhibitor piroxicam (10^−5^ mol L^−1^) significantly reduced the frequency to 0.65 ± 0.11 GCs min^−1^ (††*P* < 0.01 vs first period), although it remained significantly higher than the basal frequency (*P < 0.05 vs basal period) (Fig. 1a and c). However, the mean amplitude was not reduced by piroxicam in the presence of TTX (***P < 0.001 vs basal period, but not significantly different vs first period) (Fig. 1a and d).

In contrast, the addition of piroxicam (10^−5^ mol L^−1^) first did not change the frequency of the GCs (0.39 ± 0.06 GCs min^−1^ [basal] vs 0.40 ± 0.08 GCs min^−1^ [first period], *n* = 6; Fig. 1b and c). Nor did the addition of piroxicam first change the amplitude of the GCs (29.4 ± 4.7% CCh [basal] vs 31.9 ± 5.9% CCh [first period], *n* = 6; Fig. 1b, d). The subsequent addition of TTX (10^−6^ mol L^−1^) transiently increased the GC frequency (Fig. 1b), which then increased continuously to 0.67 ± 0.08 GCs min^−1^ (*P < 0.05 vs basal period and †*P* < 0.05 vs first period, *n* = 6; Fig. 1b and c). The addition of TTX in the presence of piroxicam significantly increased the amplitude of the GCs to 44.4 ± 5.7% CCh (**P < 0.01 vs basal period and †*P* < 0.05 vs first period, *n* = 6; Fig. 1b and d).

### Time courses of GC frequencies and amplitudes and the concentration-dependent inhibition of GCs by the cumulative addition of PGE_2_ in the presence of TTX and piroxicam

In the presence of TTX and piroxicam, DMSO (the vehicle control, 15 µL each) or PGE_2_ (10^−11^, 3 × 10^−11^, 10^−10^ …03 × 10^−7^ mol L^−1^) was added cumulatively to the bathing solution at 30-min intervals. In the vehicle control sample, the frequency of GCs decreased in a linear manner for about the first 3 h, reaching about 60% of the basal frequency, after which the rate of decline became gentler (Fig. 2a and Additional file 1: Figure S1A). In contrast, the amplitude of the GCs gradually increased for the first 3 h, reaching about 160% of the basal amplitude, and then decreased gradually, reaching the basal level after a further 2 h (Fig. 2a and Additional file 1: Figure S1B).

With the cumulative addition of the lower concentrations of PGE_2_ (10^−11^ to 3 × 10^−9^ mol L^−1^), there was no difference in the frequency or amplitude of the GCs after the addition of PGE_2_ or DMSO, whereas after the cumulative addition of the higher concentrations of PGE_2_ (> 10^−8^ mol L^−1^), the frequency and amplitude of the GCs decreased below those in the vehicle-treated control samples (Fig. 2a and b and Additional file 1: Fig. S1). The normalized mean frequencies and amplitudes of the GCs are shown in Fig. 2c and d. The data were fitted to the modified Hill equation (see Methods), and its parameters are shown in Table 2.

### Effects of selective EP_1_, EP_2_, EP_3_, and EP_4_ receptor agonists on GC frequencies and amplitudes in the presence of TTX and piroxicam

**Fig. 3:**
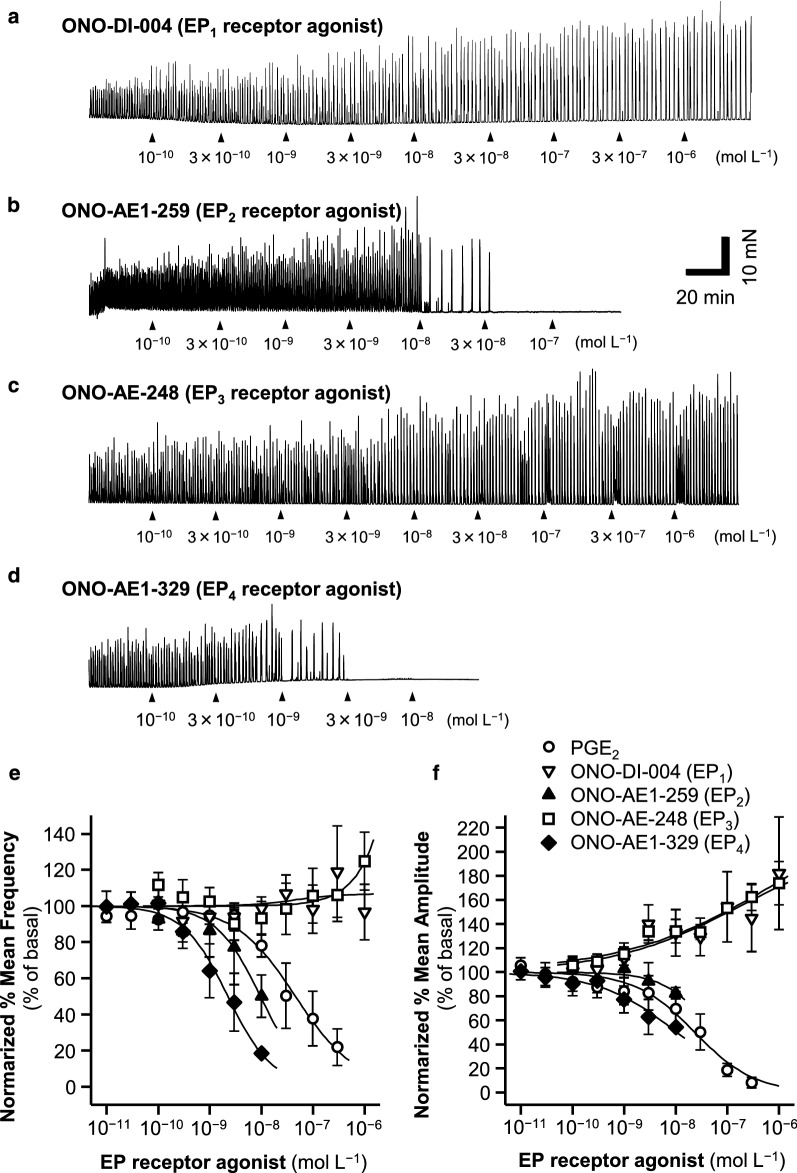
Concentration-dependent effects of selective EP_1_, EP_2_, EP_3_, and EP_4_ receptor agonists on GC frequency and amplitude. In the presence of TTX and piroxicam, vehicle control (DMSO) or each EP receptor agonist, including ONO-DI-004 (**a**; EP_1_), ONO-AE1-259 (**b**; EP_2_), ONO-AE-248 (**c**; EP_3_) and ONO-AE1-329 (**d**; EP_4_), was added to the bathing solution (10^−11^, 10^−10^, 10^−8^, 10^−7^, or 3 × 10^−6^ mol L^−1^) at ~ 30-min intervals, and these traces are shown. Concentration–response curves of the normalized mean frequencies and amplitudes (see Methods and Results) are shown in **e** and **f** with data on PGE_2_. Data are expressed as means ± SE (for frequency, PGE_2_, *n* = 4; the other agonists, *n* = 5)

Four selective EP receptor agonists, ONO-DI-004 (EP_1_), ONO-AE1-259 (EP_2_), ONO-AE-248 (EP_3_), and ONO-AE1-329 (EP_4_) (Table 1), were cumulatively added to the bathing solution (10^−10^, 3 × 10^−10^, 10^−9^ mol L^−1^ … until the GCs were abolished) in 30-min intervals in the presence of TTX and piroxicam. With the cumulative addition of ONO-DI-004 (EP_1_) and ONO-AE-248 (EP_3_), the normalized % mean frequencies of GCs did not change significantly, but the normalized % mean amplitude gradually increased to approximately 180% of the basal value (Fig. 3a, c, e and f).

By contrast, with the cumulative addition of ONO-AE1-259 (EP_2_) or ONO-AE1-329 (EP_4_), the normalized % mean frequencies and amplitudes were concentration-dependently reduced, as shown in Fig. 3 and Table 3. At concentrations of ONO-AE1-259 or ONO-AE1-329 > 10^−8^ mol L^−1^, the GCs were abolished. Because the GCs were abolished before the inhibitors reached their IC_50_ concentrations, only the IC_50_ values for the GC amplitudes were determined (> 10^−8^ mol L^−1^) (Table 3).

**Table 3:** Pharmacological parameters of inhibitory effects of PGE_2_ and each EP receptor agonist on mean frequency and mean amplitude of GCs in the CM of rat middle colon

	Mean frequency (% of basal)			Mean amplitude (% of basal)		
	IC_50_ (mol L^−1^)	*n*_H_	*R*^2^	IC_50_ (mol L^−1^)	*n*_H_	*R*^2^
PGE_2_ (endogenous agonist)	4.58 × 10^−8^	0.739	0.966	2.36 × 10^−8^	0.749	0.969
ONO-DI-004 (EP_1_ agonist)	No inhibitory effect			No inhibitory effect		
ONO-AE1-259 (EP_2_ agonist)	9.57 × 10^−9^	1.000	0.786	> 10^−8^	-	-
ONO-AE-248 (EP_3_ agonist)	No inhibitory effect			No inhibitory effect		
ONO-AE1-329 (EP_4_ agonist)	2.24 × 10^−9^	1.001	0.983	> 10^−8^	-	-

### Effects of EP_1_/EP_2_ receptor antagonist, AH6809, and selective EP_4_ receptor agonist, ONO-AE3-208, on the inhibitory response to the cumulative addition of PGE_2_ in the presence of TTX and piroxicam

**Fig. 4:**
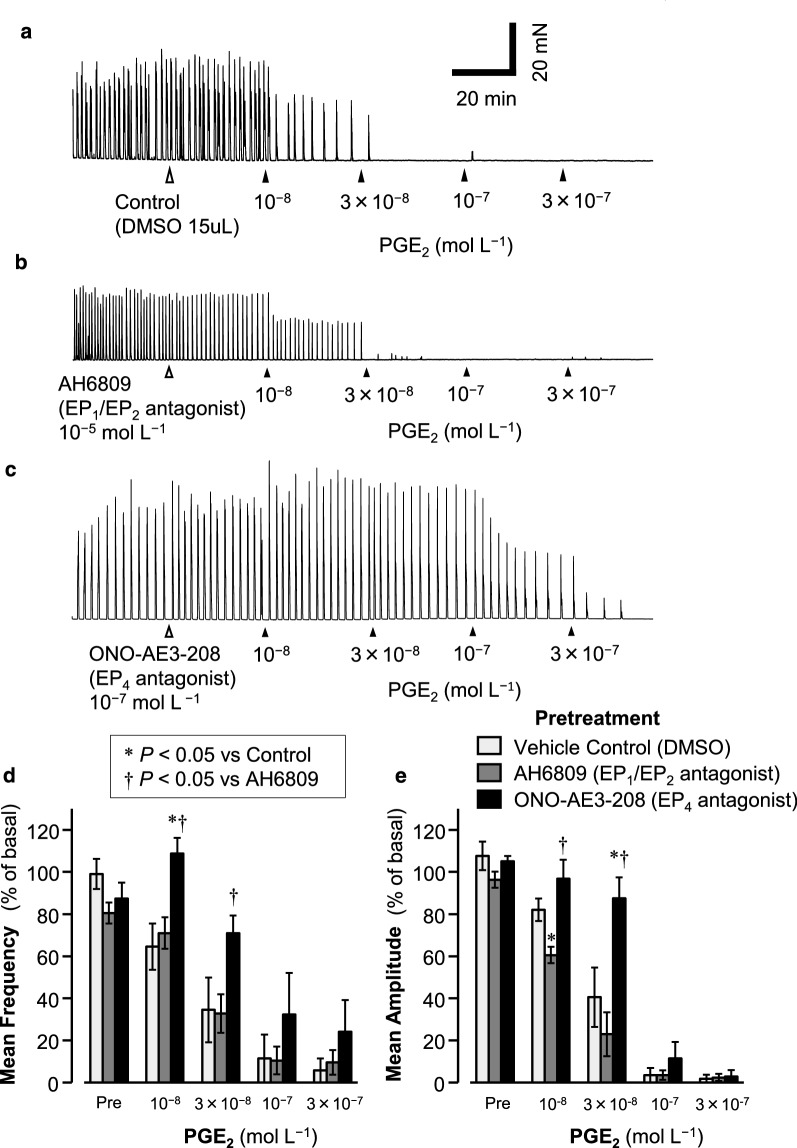
Effects of the selective EP_4_ receptor agonist ONO-AE3-208 and the EP_1_/EP_2_ receptor agonist AH6809 on the PGE_2_-evoked inhibitory response in GC frequency and amplitude. In the presence of TTX and piroxicam, AH6809 (10^−5^ mol L^−1^), an antagonist of both EP_1_ and EP_2_ receptors, ONO-AE3-208 (10^−7^ mol L^−1^), a selective EP_4_ receptor agonist, or DMSO (15 µL), the vehicle control, was added to the bathing solution. After 30 min, PGE_2_ (10^−8^ to 3 × 10^−7^ mol L^−1^) was cumulatively added at 30-min intervals. Representative traces of the PGE_2_-evoked inhibitory effect on GCs are shown for the control (**a**), AH6809-pretreated (**b**), and ONO-AE3-208-treated samples (**c**). Concentration–response curves of the GC frequencies and amplitudes are shown in **d** and **e**, respectively. Data are expressed as means ± SE (*n* = 5). *P < 0.05 was significantly different vs control, †*P* < 0.05 vs AH6809, by *t* test with Holm’s correction

In the presence of TTX and piroxicam, either AH6809 (10^−5^ mol L^−1^), an antagonist of both EP_1_ and EP_2_ receptors, ONO-AE3-208 (10^−7^ mol L^−1^), a selective EP_4_ receptor antagonist (Table 1), or DMSO (15 µL), the vehicle control, was added to the bathing solution. After the addition of AH6809, ONO-AE3-208, or DMSO, there were no significant differences in the GC frequencies or amplitudes among the groups (Fig. 4). However, the subsequent cumulative addition of PGE_2_ (10^−8^ to 3 × 10^−7^ mol L^−1^) concentration-dependently reduced the GC frequencies and amplitudes in all the groups. In the ONO-AE3-208-pretreated group, the GC frequency after treatment with 10^−8^ mol L^−1^ PGE_2_ was significantly greater than that in the control or the AH6809-pretreated group (*P < 0.05; Fig. 4d), and after treatment with 3 × 10^−8^ mol L^−1^ PGE_2_, the GC frequency was significantly greater than that in the AH6809-pretreated group (†*P* < 0.05; Fig. 4d), but not in the control group (*P* = 0.209; Fig. 4d). However, the GC amplitude after the addition of 10^−8^ mol L^−1^ PGE_2_ was significantly greater than that in the AH6809-pretreated group (†*P* < 0.05; Fig. 4e), but not compared with that in the control group (*P* = 0.190; Fig. 4e). In the AH6809-pretreated group, the GC amplitude after the addition of 10^−8^ mol L^−1^ PGE_2_ was significantly lower than that in the control group (*P < 0.05; Fig. 4e). After the addition of 3 × 10^−8^ mol L^−1^ PGE_2_, the GC amplitude was significantly larger than that in either the control (* *P* < 0.05; Fig. 4e) or the AH6809-pretreated group (†*P* < 0.05; Fig. 4e).

### Immunohistochemistry

**Fig. 5:**
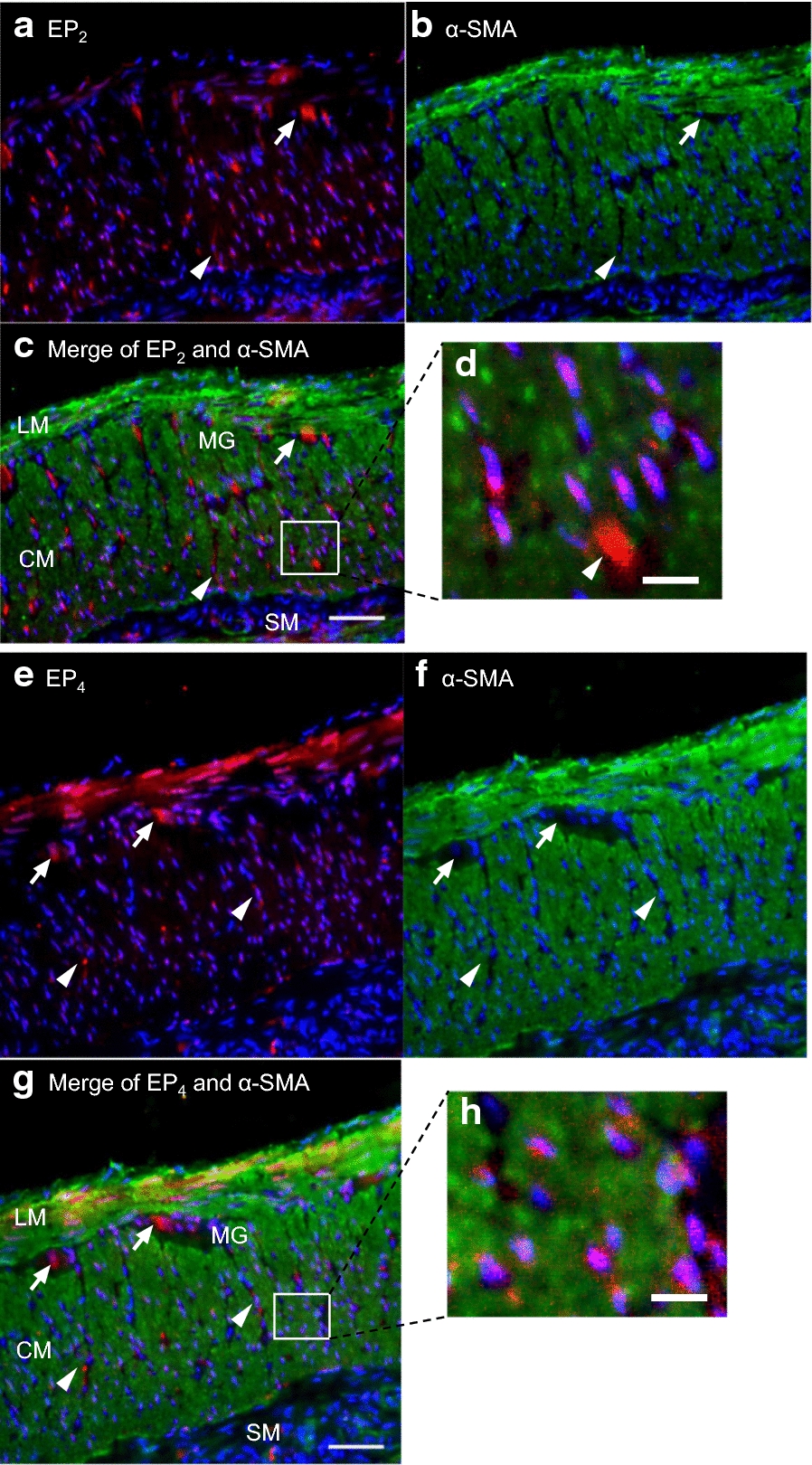
EP_2_ and EP_4_ receptor immunoreactivities in the smooth muscle layer of the rat middle colon. Immunoreactivity for the EP_2_ receptor (**a**, *red*) and α smooth muscle actin (α-SMA) (**b**, *green*) in cryostat sections (10 µm thick) of rat middle colon was visualized with fluorescence microscopy and an image acquisition system. Merged image of EP_2_ and α-SMA is shown in **c** (*bar* = 50 µm), and a magnified image of the square site is shown in **d** (*bar* = 10 µm). Immunoreactivity for the EP_4_ receptor (**e**, *red*) and α-SMA (**f**, *green*) was also visualized. Merged image of EP_4_ and α-SMA is shown in **g** (*bar* = 50 µm), and magnified image of square site is shown in **h** (*bar* = 10 µm). *Arrows* indicate EP_2_ immunoreactivity or EP_4_ immunoreactivity in myenteric ganglia. *Arrowheads* indicate EP_2_ immunoreactivity or EP_4_ immunoreactivity at interstitial sites in the circular muscle layers. *LM*, longitudinal muscle; *CM*, circular muscle; and *MG*, myenteric ganglion

*EP*_*2*_* and EP*_*4*_* receptor expression in colonic smooth muscle cells.* The EP_2_ and EP_4_ receptors in the circular and longitudinal smooth muscle layers and the myenteric plexus were immunohistochemically stained. α-Smooth muscle actin was stained as a muscle marker and the cell nuclei were stained with DAPI (Fig. 5). There was little immunoreactivity for either the EP_2_ or EP_4_ receptor in the plasma membrane or cytoplasm of the smooth muscle cells, whereas these immunoreactivities were observed in the nuclear regions (Fig. 5d and h). EP_2_ and EP_4_ receptor immunoreactivities were also observed in the myenteric plexus (Fig. 5*arrows*) and at interstitial sites (Fig. 5*arrowheads*) in the smooth muscle layer.

**Fig. 6:**
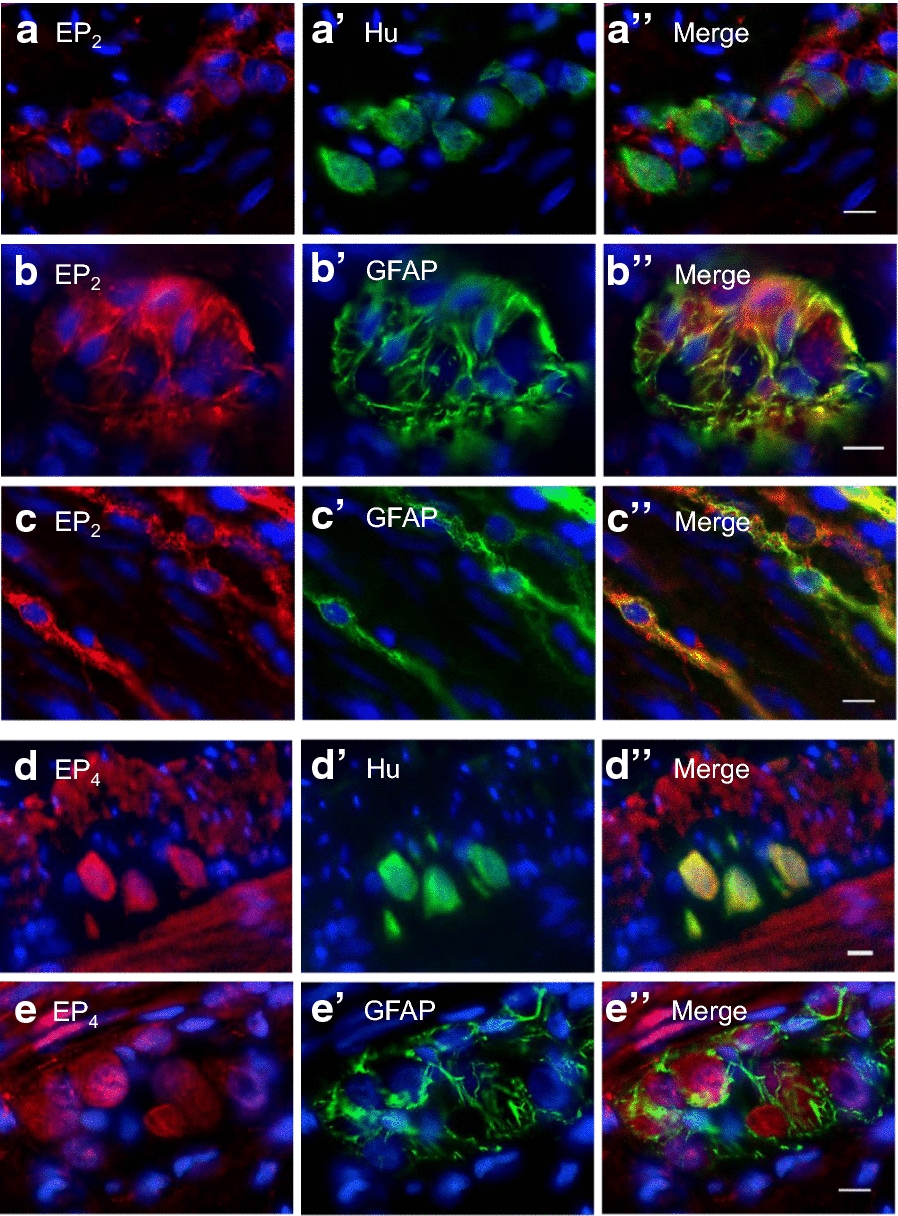
Colocalization of EP_2_ and EP_4_ receptors with neural marker HU and a glial marker GFAP in the smooth muscle layer of the rat middle colon. **a** Immunoreactivities for EP_2_ receptor (**a**, *red*) and HU (**a’**, *green*) in cryostat sections (10 µm thick) of rat middle colon. **b** and **c **Immunoreactivity for EP_2_ receptor (**b** and **c**, *red*) and GFAP (**b’** and **c’**, *green*) in a myenteric ganglion (**b**) and at an interstitial site in the CM (**c**). **d** Immunoreactivity for EP_4_ receptor (**d**, *red*) and HU (**d’**, *green*). **e**. Immunoreactivity for EP_4_ receptor (**e**, *red*) and GFAP (**e’**, *green*) in a myenteric ganglion. *Bar* = 10 µm

*EP*_*2*_* and EP*_*4*_* receptors expressed in enteroglia and enteric neurons, respectively, in the enteric nerve plexus* To determine whether the immunoreactivity for the EP_2_ and EP_4_ receptors detected in the myenteric ganglia and at interstitial sites in the smooth muscle layers occurred in the neurons or other types of cells, the tissues were doubly immunostained for these receptors, a marker of all enteric neurons, HU (embryonic lethal, abnormal vision [ELAV] protein) [21], and glial fibrillary acidic protein (GFAP). The EP_2_ receptor immunoreactivity in the myenteric ganglia had a fibrous shape and colocalized with GFAP (Fig. 6b), but not with the Hu-immunoreactive enteric neurons (Fig. 6a). However, all EP_4_ receptors colocalized with Hu (Fig. 6d), and not with GFAP (Fig. 6e).

## Discussion

This study demonstrates the separate PGE_2_-induced inhibitory effects on GC frequency and amplitude in the CM of the rat middle colon. EP_4_ receptors were predominantly involved in the inhibitory actions. The study also shows that EP_2_ receptors are expressed in the nuclear regions of smooth muscle cells and the GFAP-expressing enteroglia. EP_4_ receptors are also expressed in the nuclear regions of smooth muscle cells, but not in the enteroglia, whereas they are expressed in enteric neurons.

### Spontaneous GCs of CM in the rat middle colon are basally inhibited by enteric neural activity

The frequency and amplitude of spontaneous GCs in CM strips from the rat middle colon, lacking mucosa, were significantly (about twofold) increased by the application of TTX (Fig. 1a, c and d). This indicates that under basal conditions, enteric nerve activity potently inhibits GCs in the CM. However, the inhibition of basal PG production by treatment with piroxicam did not change the basal frequency or amplitude of the GCs (Fig. 1b, c and d). However, with neural blockade in the presence of TTX, piroxicam significantly reduced the frequency but not the amplitude of the GCs (Fig. 1a, c and d). This suggests that the PGs produced under basal conditions potentially increase the GC frequency via a nonneuronal mechanism, but that this increase is masked by the basal neural inhibitory effect.

We have previously reported that the frequency and amplitude of the GCs in LM under basal conditions were 5.3 GCs/20 min (0.26 GCs min^−1^) and 27.3% CCh (isotonic displacement contractions), respectively, and were completely abolished under PG-free conditions [18]. In the present study, the frequency and amplitude of GC in CM were determined to be 0.39 GCs min^−1^ and 25.1–29.4% CCh (isometric force contractions), respectively (Fig. 1). This suggests that the GC frequency in CM is faster than the GC frequency in LM under basal conditions. Although the isotonic contractions of LM and the isometric contractions of CM cannot be easily compared, the % ratio of the GC amplitude for CCh (10^−5^ mol L^−1^)-evoked contractions is considered to be almost the same in both cases.

### Spontaneous GCs in CM are inhibited by high concentrations (> 10^−8^ mol L^−1^) of PGE_2_

In the presence of TTX and piroxicam, the frequency and amplitude of GCs were concentration-dependently inhibited by the addition of > 10^−8^ mol L^−1^ PGE_2_ (Fig. 2). We have previously estimated that the physiological concentration of PGs in the colonic tissue is < 10^−8^ mol L^−1^ [18, 22], and have shown that this is the threshold concentration for the generation of GCs in the LM [18]. Therefore, we suggested that in the presence of PGE_2_ > 10^−8^ mol L^−1^, for example under inflammatory conditions, the mode of spontaneous GCs in the colon might change from the isometric contractions of CM to the isotonic contractions of LM.

### Identifications of EP receptors that mediate the inhibitory effects of PGE_2_ on the frequency and amplitude of spontaneous GCs

The cumulative addition of ONO-AE1-259 (EP_2_ receptor agonist) or ONO-AE1-329 (EP_4_ receptor agonist), but not ONO-DI-004 (EP_1_ receptor agonist) or ONO-AE-248 (EP_3_ receptor agonist) in the presence of TTX and piroxicam concentration-dependently attenuated the GC frequency and amplitude, and ONO-AE1-259 or ONO-AE1-329 at concentrations > 10^−8^ mol^−1^ abolished the GCs (Fig. 3). These results suggest that the PGE_2_-induced inhibitory effects on the GC frequency and amplitude are mediated by the EP_2_ and/or EP_4_ receptors, and that threshold levels of EP_2_ and EP_4_ receptor activation are required to abolish the GCs. The IC_50_ values for the inhibitory effects of ONO-AE1-259 and ONO-AE1-329 on the GC frequency (Table 3) in the presence of TTX and piroxicam were similar to the *K*_i_ values for the EP_2_ and EP_4_ receptors (Table 1), respectively, but the order of potency was ONO-AE1-329 (EP_4_) > ONO-AE1-259 (EP_2_) (Table 3). Furthermore, the Hill constants for ONO-AE1-259 and ONO-AE1-329 for their effects on the GC frequency were almost 1 (Table 3). These results suggest that these selective-receptor-evoked reductions in the GC frequency and amplitude are attributable to the activation of the EP_2_ and EP_4_ receptors.

Pretreatment with the EP_4_ receptor antagonist ONO-AE3-208 significantly inhibited the attenuating effect of PGE_2_ at 10^−8^ mol L^−1^ on the GC frequency (Fig. 4d) and that of 3 × 10^−8^ mol L^−1^ PGE_2_ on the GC amplitude (Fig. 4e), compared with those in the control groups. Moreover, ONO-AE3-208 significantly inhibited both the 10^−8^ mol L^−1^ and 3 × 10^−8^ mol L^−1^ PGE_2_-induced attenuation of both the GC frequency and amplitude, compared with those in the groups pretreated with the EP_1_/EP_2_ receptor antagonist AH6809 (Fig. 4d and e). These results suggest that the PGE_2_-induced attenuation of the GC frequency and amplitude is predominantly attributable to the activation of the EP_4_ receptor, but not to that of the EP_2_ receptor. However, the present results have shown that EP_2_ receptor agonist ONO-AE1-259 attenuates the GC frequency and amplitude. The reason for this inconsistency remains unclear, but might be due that the *K*_i_ values of EP agonists shown in Table 1 were based on mice EP receptors. In rats, ONO-AE1-259 might have lower *K*_i_ value for EP_4_ receptors than that in mice.

It has been reported that the *K*_i_ values of PGE_2_ for the EP_2_ and EP_4_ receptors are < 10^−8^ mol L^−1^ (Table 1). However, the present study showed that the IC_50_s for the inhibitory effects of PGE_2_ on the GC frequency and amplitude were > 10^−8^ mol L^−1^ (Table 3). Moreover, the Hill constant (*n*_H_) for the PGE_2_-induced reduction in frequency was < 1 (Table 3), indicating negative cooperativity. In LM, PGE_2_ is reported to activate the EP_1_ and EP_3_ receptors, generating and enhancing spontaneous GC-like contractions [18]. It is therefore suggested that the PGE_2_-induced reduction in the GCs is mediated by the activation of the EP_4_ receptor and that this inhibitory effect is negatively affected by the activation of the EP_1_ and/or EP_3_ receptors by PGE_2_. This might be because PGE_2_ not only activates the EP_4_ receptor, but also activates the EP_1_ and EP_3_ receptors, which are considered to enhance GCs.

Pretreatment with AH6809 did not affect the PGE_2_-induced attenuation of the GC frequency compared with that of the vehicle control, but in the presence of AH6809 and at 10^−8^ mol L^−1^ PGE_2_, the GC amplitude was significantly lower than that in the vehicle control (Fig. 4e). This suggests that PGE_2_ not only attenuates the amplitude of GCs by activating EP_4_ receptor, but also partly inhibits this PGE_2_-induced attenuation by activating the EP_1_ receptor.

### Distributions of EP_2_ and EP_4_ receptors in the rat middle colonic muscle layers

In our immunohistochemical study, the EP_2_ and EP_4_ receptors were expressed at nuclear sites in smooth muscle cells (Fig. 5). These expression patterns are the same as those of the EP_1_ and EP_3_ receptors [18]. The EP_2_ receptors were also expressed in GFAP-expressing enteroglia in the myenteric ganglia and at interstitial sites in the CM layer (Fig. 6a and b), whereas the EP_4_ receptors were expressed in the somata of HU-expressing cells in the myenteric neurons (Fig. 6d and e). In the present study, spontaneous GCs were measured in the presence of TTX, so that the PGE_2_-induced attenuation of GCs is attributed to the EP_4_ receptors (predominantly) and EP_2_ receptors in the smooth muscle cells. Furthermore, the EP_1_ and EP_3_ receptors in the smooth muscle cells appear to contribute to the inhibition of the EP_2_- and EP_4_-receptor-mediated attenuation of GCs. However, in this study, we did not investigate the role of EP receptor expression in the enteric neurons and enteroglia. Therefore, the functions of PGs in the myenteric neurons and enteroglia must be investigated in future studies.

### Physiological role of PGE_2_-regulated spontaneous GCs

In our previous study [18], the physiological concentration of PGE_2_ in the intestinal tissues was estimated to be between 10^−9^ and 10^−8^ mol L^−1^, and no spontaneous GCs of LM (LMGCs) were observed when endogenous PG production was abolished with piroxicam. However, when the intestinal tissues were treated with exogenous PGE_2_ above the threshold concentration (10^−8^ mol L^−1^), LMCGs were generated and enhanced in a PGE_2_-concentration-dependent manner, mediated predominantly by smooth muscle EP_3_ receptors [18]. In contrast, in the present study, we have shown that in the presence of piroxicam, the GCs of CM (CMGCs) were the same as under basal conditions (Fig. 1b). However, when the CM was treated with > 10^−8^ mol L^−1^ PGE_2_, the CMGCs were attenuated in a concentration-dependent manner, mediated predominantly by smooth muscle EP_4_ receptors (Fig. 2). This suggests that colonic motility is set to a CMGC-generating (not LMGC-generating) mode at PGE_2_ concentrations below the basal physiological condition (< 10^−8^ mol L^−1^), but the mode changes to a LMGC (and low CMGC)-generating mode at prepathological/pathophysiological levels of PGE_2_ (> 10^−8^ mol L^−1^) when the tissue level of PGE_2_ is increased. The intestinal PGE_2_ level is considered to increase under a variety of physiological and pathophysiological conditions, including mechanical stimuli, such as the stretching of intestinal segments [23, 24]; chemical stimuli, such as short-chain fatty acids [25]; and acute inflammatory injury [26]. Moreover, > 10^−7^ mol L^−1^ PGE_2_ is considered to be pathophysiological [27]. It has been reported that in the colonic mucosa, > 10^−8^ mol L^−1^ PGE_2_ induces transepithelial Cl^−^ secretion, inducing fluid secretion in the guinea pig [28], human [29], and rat [30] colon. Therefore, adequate transepithelial fluid secretion is considered to occur together with both CMGCs and LMGCs in physiological high concentrations of PGE_2_, and massive fluid secretion occurs together with only LMGCs but not CMGCs in pathophysiological higher concentrations of PGE_2_.

## Conclusion

In conclusion, the present study suggests that at high but still physiological concentrations of PGE_2_ (approximately 10^−8^ mol L^−1^), the lubricated colonic luminal contents are smoothly transported with both CMGCs and LMGCs, but at a much higher prepathological/pathophysiological concentrations of PGE_2_ (> 10^−7^ mol L^−1^), the highly fluid contents are promptly flushed out with only LMGCs, inducing secretory diarrhea.

### Supplementary Information

**Additional file 1: Figure S1.** Unnormalized time-courses of the % mean frequencies and amplitudes of GCs to the frequencies and amplitudes just before the first additions of vehicle (DMSO) and PGE_2_.

## Data Availability

Not applicable.
