# Supplementary figures and images for: Role of PGE2 in colonic motility: PGE2 attenuates spontaneous contractions of circular smooth muscle via EP4 receptors in the rat colon
Source: J Physiol Sci. 2021 Feb 23;71:8. doi: 10.1186/s12576-021-00791-4 (PMC10717948; doi:10.1186/s12576-021-00791-4)

Supplemental Figure 1

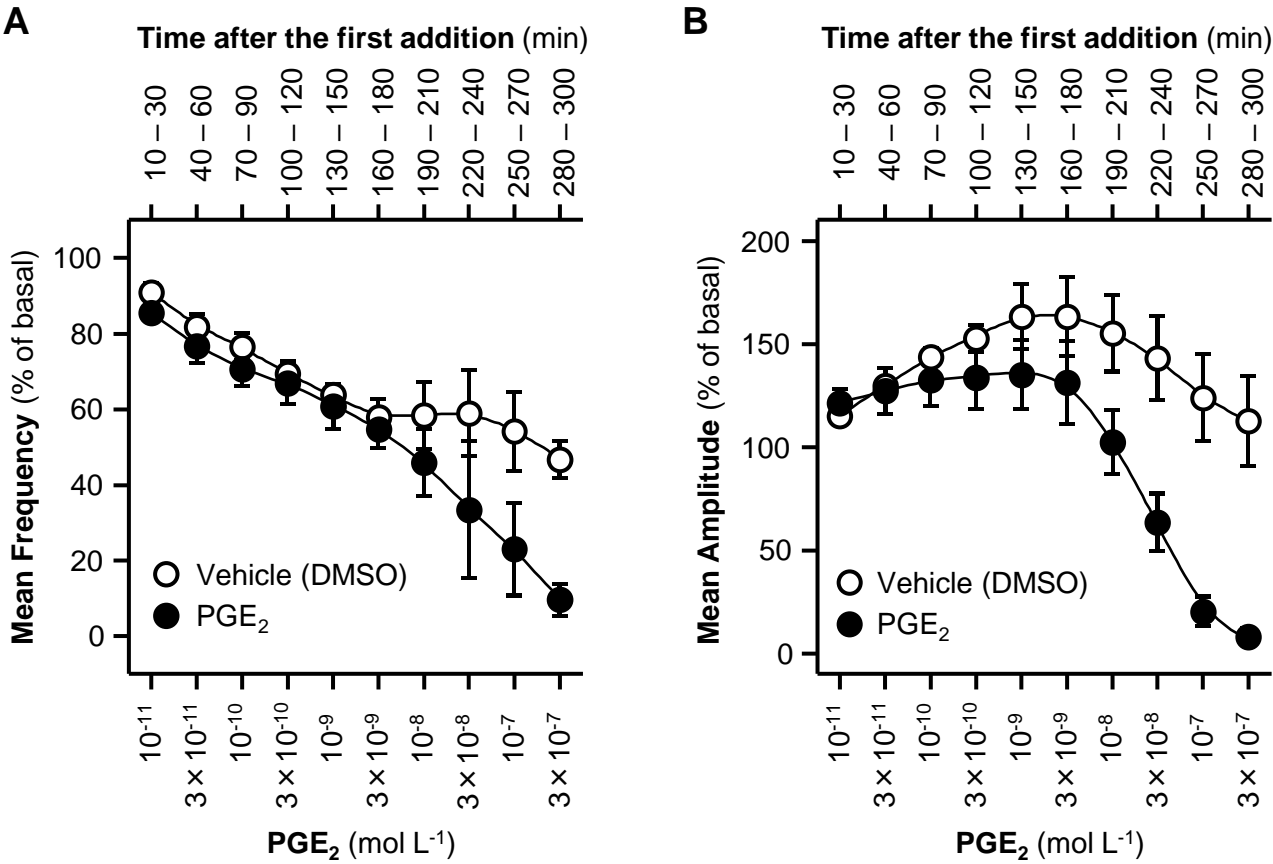

Supplement: Supplementary file 1 — Additional file 1: Figure S1. Unnormalized time-courses of the % mean frequencies and amplitudes of GCs to the frequencies and amplitudes just before the first additions of vehicle (DMSO) and PGE2. [file 12576_2021_791_MOESM1_ESM.pdf]
